# Emergent Guiding Principles for STEM Education

**DOI:** 10.1007/978-3-030-58948-6_6

**Published:** 2021-03-12

**Authors:** Lawrence C. Ragan, Lorraine J. Ramirez Villarin

**Affiliations:** 1 Pennsylvania State University, Altoona, PA USA; 2 Valdosta State University, Valdosta, GA USA; 3 Ragan Education, State College, PA USA; 4 University of North Georgia, Dahlonega, GA USA

## Abstract

This chapter highlights a series of guiding principles that emerged from the information collected and collated following the X-FILEs Workshop. The guiding principles were developed from themes that appeared in multiple formats and across most or all technology categories. These propositions can inform and serve as the foundation for the design and development of future STEM education. These nine guiding principles, divided into four clusters, emphasize a learning environment that is student-engaged, flexible, and fluid, provides equitable access and accessibility to all, and is personalized to the learner, co-contributed and multiply-sourced, safe and secure, and ethical.

## Motivation

STEM-related jobs are on the rise. Workers are required to exercise critical thinking and decision-making skills while being knowledgeable and competent in domains related to Science, Technology, Engineering, and Math (STEM). The eXploring the Future of Innovative Learning Environments (X-FILEs) Workshop, hosted by the Florida Institute of Technology in November of 2018, allowed stakeholders to make informed decisions about the adoption and use of innovative learning environments (ILEs) in higher STEM education. Participants had the opportunity to consider four emerging technologies that could assist in this effort: personalized and adaptive learning, multimodal learning formats, cross reality (XR), and artificial intelligence and machine learning. The research team gathered shared ideas through online meetings and collaborative activities that reflect on the opportunities and challenges to expect while implementing ILEs in higher education STEM curricula.

Since these immersive technologies are quickly evolving, it has been challenging for institutes to implement integrated STEM education programs that utilize them (Blackley and Howell 2015; Briener et al. 2012). Therefore, these guiding principles are a good starting point.

## Recurring Themes

A series of recurring themes emerged from the data collected at the 2018 workshop. These themes appeared across most or all four technology categories and served as the basis for the development of guiding principles for the future of STEM education. In most, if not all cases, the identified themes were interconnected and related to each other. For example, providing students a personalized learning environment based on personal interest and preferences is dependent upon having collected data about each student. In collecting data on student preferences, concern for student rights to privacy must be evaluated.

The nine emerging principles were classified into four clusters to better understand their correlation and importance within the academic instructional design field. The four clusters encompass the student experience, the learning community, its availability, and the learners’ protection. While considering the affordances of the supporting technologies in the generation of ILEs, the limitations of these technologies must also be weighed.

### Student Experience

#### Student-Engaged Learning Environment

While the lecture format of instruction has been the norm in higher education for years, it is time for the students to play a more active role in their educational experience. STEM education requires learners to solve real-world challenges through the application of scientific and mathematical principles, the engineering process, and the use of current and emerging technologies. To help retain learners who choose STEM programs in college, active learning approaches are a must (Olson and Riordan 2012). Carefully crafted ILEs allow students to construct representations of their own knowledge and practice applying their skills to new and novel problem scenarios. Donally (2018) suggests students create monuments for a history project, design worlds with unique ecosystems for science, or bring a literary reference to their reality for a reading assignment.

Personalized learning provides the opportunity to tailor instruction to the individual’s needs, skills, goals, and learning preferences while consistently monitoring progress (Sampson 2001, cited in Sampson et al. 2002). This approach promotes improvement while enabling participants to take control of their learning. Nonetheless, student-centered environments like multimodal learning require students to have a high degree of agency and metacognition to recognize when learning is happening and when to challenge themselves (Bezemer and Kress 2016; Moreno and Mayer 2007; Phuong et al. 2017; Sankey et al. 2010).

Virtual reality provides an opportunity to keep the student as the center of the learning environment. While being “mistake-free” (Bailenson 2018), participants actively explore virtual worlds or encounter scenarios where learning takes place through trial and error. Cross reality experiences like these have shown equivalent learning gains (Madden et al. 2018; Winkelmann et al. 2017) or lesser learning gains than traditional instruction (Makransky et al. 2019). However, cross reality experiences focus more on scientific thinking processes and behaviors than it does in content knowledge.

Applications of artificial intelligence help craft these learning environments through automated tutoring, personalized learning, student knowledge assessment, and automating mundane instructors’ tasks (Lu and Harris 2018). Devices like chatbots and auto-graders enable progress monitoring which may pinpoint strengths and weaknesses that can steer students and instructors on the proper academic path.

#### Personalized Learning

Students do not learn in the same way. They learn better when instruction is individualized to the learner’s needs (Kerr 2016). However, factors like academic background, culture, language, or disability may influence the manner an individual learns. Having the advantage of a personalized or customized learning environment can be beneficial for students, as well as instructors. Dashboards that monitor individual activity, provide feedback, alert instructors of students’ difficulties, and suggest other approaches are likely to enrich instruction. For example, adaptive learning environments are based on individual students’ profiles created upon their strengths, weaknesses, and pace of learning (Becker et al. 2018). The technology tracks progress and adjusts the learning path enabling instructors to deliver timely feedback. These adaptive learning systems promote self-regulated learning which has been shown to be a predictor of academic success (Boekaerts 1997; Miltiadou and Savenye 2003; Pintrich and De Groot 1990; Rosen et al. 2010). A study by Prain et al. (2013) reported that math students participating in a personalized curriculum got better scores and were more engaged than those who were not.

A “one size fits all” curriculum may not fulfill everyone’s needs (Gee 1996; Phuong et al. 2017). While studying augmented reality applications for cross reality, one recommendation made by Radu (2014) was that experiences be designed based on curriculum and pedagogical needs while providing the opportunity for faculty to customize them accordingly. For example, an AI-driven personalized learning system would adjust the context of the content based on the learner’s interest and preferred learning modality while still meeting desired learning outcomes.

In adaptive learning systems, students work at their own pace. Since learner activities are auto-graded, they can receive feedback and scaffolding when needed. It is important that the student does not feel isolated. Communication should not be a concern between students and instructors. As in any learning experience, support should be available at any time in the form of live instructors or automated systems to provide guidance or answer questions.

#### Flexible, Fluid, and Evolutionary

Current and emerging technologies are transforming constantly tending to learners’ and instructors’ needs while also considering academic institutions’ demands. The required technology for most of these ILEs can already be found in many homes, classrooms, and workplaces in the form of smartphones, computers, or game systems. Bailenson (2018: 9) predicts virtual reality to be “a mainstream technology, worth an estimated $60 billion, in the next decade.” As for augmented reality, it is expected to reach $60 billion in 2020 (Porter and Heppelmann 2017). This flexibility of access provides the opportunity for informal learning environments almost anywhere. Multiuser 3D virtual world environments allow geographically dispersed learners to participate in a traditional classroom-like environment while learning at their own pace (Olasoji and Henderson-Begg 2010) and in their own time zone. This provides instructors the opportunity to convey fluid and seamless learning experiences inside and outside the classroom.

The flexibility of content and context is another advantage of ILEs. Having the malleability to enhance content by presenting it in more than one sensory mode or incorporating text-based information and multimedia using a diversity of modes can generate multiple access points for learning (Bezemer and Kress 2016; Matusiak 2013; Nouri 2018; Sankey et al. 2010) as those provided through multimodal formats. Well-designed courses like those for adaptive learning foster the learning of processes and strategies that promote self-regulation in students (Dabbagh and Kitsantas 2013; Hense and Mandl 2012; Shea and Bidjerano 2009). On the other hand, higher interactive virtual environments work through learning activities as immersion stimulates emotional involvement which is essential in the relocation of learning to long-term memory (Aldrich 2009).

For measuring student progress and performance, formative and summative assessments inform the student of advancement toward the learning objectives and provide a final grade of achievement. However, many methods do not objectively address student achievement or translate into real-world outcomes in STEM fields (Spector 2017). Using “real-to-life” and situated learning can enable students to practice or be measured within the context of the domain. Simulations, virtual reality, and augmented reality can recreate “real-world” experiential opportunities for learning that would otherwise be impossible in a regular classroom. These immersive environments promote learning without any hazards to students or others. For example, surgical procedures may be practiced safely as no “real” patients will be in danger (Health Scholars n.d.). Chemicals can be mistakenly combined or spilled and cleaned up by pressing a recycle button (Faulconer and Gruss 2018) with no harm to participants. These experiences can be paused, reset, and available on-demand (Lynch and Ghergulescu 2017), while the dynamic nature of the computer system records and gathers data in the background as the learner moves along (Rose 1995). Learner activities like these bring relevance to context while replicating many tasks professionally encountered in STEM-related fields.

STEM educators need to be open to a variety of technologies and recognize the interoperability of various systems. Learning management systems (LMS) may not provide the instructor with all they need to support a synchronous or asynchronous learning system. In some cases, there are add-ons to the LMS that may be used to expand the system’s functionality. Social media platforms, cross-platform messaging applications, and other application software may facilitate instruction even if these programs’ main duty is not related to academics. For example, Twitter can be used to promote interaction among peers and instructors, while Facebook and WhatsApp are flexible enough to create groups or chats for which you can share video and audio recordings and get immediate feedback.

### Learning Community

#### Include Both Individual and Group Interactions and Input

Students who select STEM programs in college might be looking forward to engaging in active learning approaches. To challenge these learners, instructors may want to alternate between some of these technologies for individual and collaborative tasks. For example, computer-supported collaborative work assesses individual and collaborative potentials based on individual and shared contributions. Ward and Sonneborn (2009) indicate that creative problem-solving in groups must consider the learner group contribution and the quality and quantity of ideas produced through individual collaborations. Other communication systems like collaborative virtual environments allow users to share a three-dimensional digital space while occupying remote physical locations (Yee and Bailenson 2006). With dozens of multiuser experiences available (JuegoStudios 2019) like social virtual reality instruction, facilitated discussion, and project collaboration, instructors and researchers have the freedom to incorporate these for either learning or research purposes.

As for personalized and adaptive learning, interactions occur between student and content and between student and instructor. Student-student interactions are not typical. Moore (1989: 1) describes student-content interaction as that which “results in changes in the learner’s understanding, the learner’s perspective, or the cognitive structures of the learner’s mind.” Student-instructor interaction, often based on performance, usually takes place through the learning management system.

Besides promoting collaborative learning, interactions that can foster communication skills and cultural enrichment could enhance these learning experiences. Donally (2018) claims that augmented reality, virtual reality, and mixed reality allow students to shape how they view others around the world through interaction and collaboration. It should be a top priority for institutions with access to these cross reality resources to have the opportunity to stimulate interaction and promote collaboration while inviting students from other academic institutions with similar access. These immersive technologies should expand to engage global interactions and feedback.

Multimodal learning environments facilitate interaction since those who might have trouble communicating in one mode can interact in another. For example, students can arrange to connect with culturally diverse students who might be well-versed in the digital world or other multilingual or English language learners or others who have similar learning disabilities, creating a rich, comfortable learning experience. Considering the evolving demographics of students expected in higher education, multimodal formats can help customize student-centered experiences and address diversity issues.

#### Co-contributed and Multiply Sourced

Increasingly, with access to sophisticated online content development tools, the instructional material used in a course may be generated from multiple sources. Students, generating content through social media or self-publishing, perceive a class assignment as an opportunity to create, generate new information, or assimilate existing data into new perspectives. This student-generated content makes an increasingly critical contribution to the course experience. Indeed, with the trend toward active and engaged learning, students are being encouraged and directed to create new works, products, and ideas that may have added value outside of the course.

The content for today’s classes is unlikely to be singularly sourced, that is, comes from a single text or course pack. Content from multiple sources can be presented in different modes (e.g., gestures, visuals, multimedia, text-based information) (Bezemer and Kress 2016; Matusiak 2013; Nouri 2018; Sankey et al. 2010) and greatly enhance the learning experience. The richness of content digitally accessible is extensive and more likely to be integrated into the learning materials than other reference sources. In many cases, live data streams provide the most current data source. For example, assembling a virtual expert panel of industry leaders directly linked into a live lecture can further approximate reality and enrich the experience for all participants. With this access to a plethora of content comes the responsibility to ensure the accuracy and validity of the information. This alone becomes a new skill the lifelong learner will need to master!

### Availability

#### Equity of Access

As new learning systems emerge, the concern of equitable access for all learners needs to be reviewed. These ILEs are crucial learning instruments, and all students should be provided equal access regardless of privilege, ability, or economic situation. More sophisticated adaptive learning systems, often developed commercially, may also increase the cost for engagement for the learner. Even as the cost of cross reality devices may decrease, the technical requirements may include higher-speed bandwidth, device batteries, and add-on components. In some locations, for example, broadband access may limit student interaction with cross reality systems.

Technologies such as artificial intelligence can require resources from machine learning models for each assessment and grade level (Balfour 2013; Shermis et al. 2010; Nehm et al. 2012). Curating learning environments for different modes can also be quite complex and demand additional allocation of resources (Bezemer and Kress 2016). Add to this finding the preparation of the physical space including enhanced lighting and audio, upkeep of devices, and the time and money it takes to train instructors and technicians. This will make it challenging for certain academic institutions who lack the resources. A digital divide still exists between certain educational institutions and communities (ACT Center for Equity in Learning 2018).

#### Accessibility

Assistive technology refers to any item, piece of equipment, software program, or product that increases, maintains, or improves the functional capabilities of people with disabilities and is in the form of equipment, software, or hardware (Assistive Technology Industry Association 2019). Immersive learning environments such as those made possible through virtual worlds simulations can provide participants with a sense of physical movement which can enhance their experience.

This same advantage, however, in some circumstances, may deprive an individual with disabilities from this enhanced experience (Ryan 2019). The Americans with Disabilities Act prohibits discrimination toward this population by allowing support in the workplace and academic institutions (U.S. Equal Employment Opportunity Commission n.d.; U.S. General Services Administration 2018). Unfortunately, most personal devices, nowadays, do not accommodate the emerging technological demands for replicating experiences for this group and other diverse learners. Therefore, engagement will be interrupted as you exit the classroom. Providing this population with the same opportunities as traditional student has is not only keeping compliant with the law but also the right thing to do to achieve equity and inclusiveness.

### Learner Protection

#### Safe and Secure

Keeping the privacy of users seemed to be a recurring concern across three of the four technologies explored. Unfortunately, the digital world is not exempt from inappropriate or unlawful actions. Users may be exposed to threats like cyberbullying, griefing, and security attacks. For example, research done by Outlaw and Duckles (2017) reflected that female participants in social virtual reality experiences had encountered “flirting, a lack of respect for personal boundaries, and socially undesirable behavior,” similar to those in real life, leaving little desire to return to these platforms. As for multiuser environments, the number of accounts and avatars that can be created can increase trust and identity concerns (Warburton 2009). In order to minimize this, proper training would be required for instructors and learners since a lack of technical skills could make them more susceptible to this kind of action. Faculty and students need to understand that some channels or modes may be “open” for public viewing. Professional development can provide orientation on what university tools are available to ensure students’ safety and privacy. Nevertheless, training is time-consuming and costly.

For example, during the COVID-19 pandemic, Zoom became one of the most popular platforms for teleconferencing. However, “uninvited guests” would ruin the video conference experience using shocking imagery, racial slurs, or profanity leading to a phenomenon known as Zoom bombing (Lorenz and Alba 2020). These “trolls” (disruptive, uninvited strangers) were hard to identify and, therefore, suffered little to no consequences for their malicious actions. Zoom implemented stricter measures of access control enabling the host to have more restrictions over the meeting. Nonetheless, these incidents may still happen in other platforms and without the user’s knowledge. For example, spyware can be installed remotely through an email, a photo download, or an instant message. This type of malware records screenshots of information typed (e.g., passwords, usernames), and media accessed.

Access control measures can be a challenge, but there are certain options that can improve security. More and more organizations are adopting multifactor authentication which requires users to use more than one physical device to verify their identity. The second factor device can be your mobile phone in addition to a personal computer. Facial recognition and fingerprint readers are also common biometrics mechanisms used for identity authentication. Other options include the power to access your information from another device which gives you the flexibility to receive notifications when changes are made in your accounts. To protect your information, data encryption allows the system to scramble the data and share a secret key or key pair with those who access it. However, as technology changes every day, access control measures need to evolve to outsmart attackers.

#### Ethics

The increasing technical dimensions of developing STEM curriculum demands a critical review of the ethical aspects of the use of these tools in future educational systems. As presented previously, the safety and privacy of each learner are paramount. Although proper netiquette is expected among users, hacking and cyberbullying are dangers to which everyone is exposed. As users create multiple accounts and avatars, for example, in a multiuser environment, trust and identity may be threatened (Warburton 2009). These hazards can be intimidating for some and may limit use especially if an individual is not familiar with the different technologies. To minimize falling into these negative experiences, rigorous training on the different modes and technologies is a must for instructors and learners going through this digital transition. However, this may not be enough. Certain corporations, like Microsoft, are adding face-to-face interactions to virtual and persistent collaboration rooms to enhance trust as they hope to expand to the workforce market with a cross reality platform called Hololens (Weise 2019).

As technologies involving cross reality evolve bringing features like sight, sound, smell, taste, pressure, heat, and texture to the learning environment, the danger exists in disconnecting the user from reality. Bailenson (2018: 46) indicates that “VR feels real, and its effects on us resemble the effects of real experiences. Consequently, a VR experience is often better understood *not as a media experience, but as an actual experience,* with the attendant results for our behavior.” If virtual experiences are perceived as real, for example, it raises the question of ethics in conducting research with “virtually real-life” specimens and “real-life” specimens.

Another ethical challenge relates to the way algorithms work in predicting performance. Learners might be presented with opportunities or support based on their anticipated scores or scores provided by a machine algorithm (Ogor 2007; Ciolacu et al. 2017). Users may want to “play the system” to deceitfully display to have a better performance. Instructors may feel uncomfortable trusting results from an algorithm and may resort to other forms of assessment. However, FERPA requires student information to be kept private, including scores. To keep compliant, strict rules of engagement, regulations, and set boundaries should be established for users and offenders.

The use of increasingly intrusive technologies that collect, store, analyze, and manipulate personal data presents a myriad of ethical decisions that must be addressed and defined. Although AI and machine learning has the capacity to track and adjust the course content to the interest and preferences of the learner, they also present the opportunity to negatively influence students’ behaviors. Defining the boundaries of appropriate social behavior when presented such powerful data gathering tools is a crucial step in the development of future learning systems.

## Conclusion

The guiding principles for STEM education emerging from the X-FILEs Workshop provide a foundation on which to consider the design and development of future STEM innovative learning environments. These principles are not exclusive to STEM education but are also critical to the construction of all course instruction in the near and future education systems. These guiding principles reflect the capability and promise of the technologies that make it possible as well as the challenges and threats presented by the use of these technologies.

The guiding principles emerging from the X-FILEs Workshop aggregated into four clusters representing the student experience, the learning community, availability, and learner protection. All of the clusters reflect some aspect of the learner’s response to the design of the learning system. Many of the principles are interrelated and, in some cases, represent “the other side of the coin” of a stated principle. For example, a personalized learning system described in Sect. 2.1 also presents the challenges of protecting personal data gathered from profile information of the student as referenced in Sect. 2.4.

Forefront in these principles is a focus on the changing role and experience of the learner. These principles embrace an engaged and active learner as both a recipient and a contributor to the learning system. Capitalizing on the capability for all class participants to contribute to the construction of the course content, the principle stated in Sect. 2.2, empowers students to improve and enhance the course materials. In increasing the responsibility of the learner as a co-contributor, the act of generating, analyzing, and publishing course content also prepares them for controlling and directing their own future learning opportunities.

Perhaps the most challenging ideas and unanswered questions evolve from the ideas in the Sect. 2.4 principle. The promise and power of the previous principles raise the awareness and concern for addressing how to protect the individual and group rights of learners. The unprecedented affordances and opportunities enabled by the technologies and methodologies explored in the X-FILEs research create a multitude of ethical questions regarding harvesting, analyzing, and manipulating student data and input. Where, when, and how to use student data within the learning system need to be defined and appropriate guidelines and protections established.

The guiding principles emerging from the X-FILEs Workshop can serve as the basis for the design and development of student-centered, interactive, and increasingly effective innovative learning environments. These principles need to be further vetted, refined, and improved in order to address all dimensions of the learning ecosystem. Input and responses from all stakeholders also need to be garnered in order to ensure as thorough and comprehensive of a set of guidelines as possible. The future of STEM education is exciting and creates the responsibility of doing so thoughtfully and intentionally in order to maximize learning and preparing students for lifelong learning.
